# Factors impacting referral of JIA patients to a tertiary level pediatric rheumatology center in North India: a retrospective cohort study

**DOI:** 10.1186/s12969-020-0408-4

**Published:** 2020-03-04

**Authors:** Manjari Agarwal, Caroline Freychet, Sumidha Jain, Abhay Shivpuri, Anju Singh, Veronique Dinand, Sujata Sawhney

**Affiliations:** 1 Division of Pediatric Rheumatology, Institute of Child Health, Sir Ganga Ram Hospital, New Delhi, India; 2 HESPER Laboratory, Claude-Bernard University, Lyon, France; 3 Department of Research, Sir Ganga Ram Hospital, New Delhi, India

**Keywords:** JIA, Access to care, Diagnostic delay, India

## Abstract

**Background:**

JIA studies demonstrate that there is a “window of opportunity” early in the disease course during which appropriate management improves outcomes. No data is available regarding patients’ pathway, before first pediatric rheumatology (PR) evaluation in India, a country where health-care costs are self- paid by patients and where a significant shortage of pediatric rheumatologists (PRsts) is known. This study aimed to describe time from onset of symptoms to first PR visit of JIA patients to a tertiary center in India and factors that impact this.

**Methods:**

This retrospective study is from data collected at the PR center, Sir Ganga Ram Hospital (SGRH) in New Delhi. JIA patients fulfilling ILAR 2004 criteria and seen at least twice from 1st October 2013 to 30th September 2018 were included. Data collected were: demographic details, history of disease, referral practitioner, clinical and laboratory features, treatments. Mann-Whitney U-test, Chi square and logistic regression were used as appropriate to study factors that determined time to first PR visit.

**Results:**

Five hundred and twenty patients were included: 396 were diagnosed at this PR center (group A), 124 were previously diagnosed as JIA and managed by non PRsts before first PR visit (group B). Median time from symptom onset to first PR visit was 4.1 months and median distance travelled 119.5 km. Despite ongoing treatment, group B patients had more aggressive disease and resided further away as compared to Group A patients.

On univariate analysis, factors that predicted PR visit within 3 months were private patients, short distance to travel, family history of inflammatory disease, history of fever, history of acute uveitis or high ESR. On multivariate analysis all these factors were significant except high ESR and acute uveitis.

**Conclusion:**

Time to first PR assessment at this center was comparable to that seen in western countries. Cost of care and long distance to the center delayed consultation; acuity of complaints and family history of rheumatologic condition hastened referral.

Possible solutions to improve referral to PR centers would be to increase the number of PRsts and to improve medical insurance coverage.

## Background

Juvenile idiopathic arthritis (JIA) is the most common pediatric rheumatological disorder. It is defined as an inflammatory joint disease persisting longer than 6 weeks in children under 16 years old and after exclusion of all other causes of arthritis [1]. Severe painless uveitis can be associated [2]. The worldwide prevalence is highly variable from 6 to 400 per 100,000 [3].

It has already been demonstrated that there is a “window of opportunity” early in the disease course, during which treatment can alter the natural history of the disease process [4, 5]. Early medical intervention comprising disease-modifying anti-rheumatic drugs (DMARDS), and in the past two decades biologic response modifiers (BRMs) have dramatically decreased the risk of joint and/or ocular damage [6–9]. This has been recently confirmed by the “Epidemiology, treatment, and outcome of childhood arthritis throughout the world (EPOCA) study” in which 9081 children with JIA were enrolled at 130 pediatric rheumatology (PR) centers in 49 countries: damage was associated with referral delay [10]. There are no international guidelines on the most appropriate time to referral except for the British Society for Pediatric and Adolescent Rheumatology Standards of Care (BSPAR) that advocates that children with suspected JIA should be assessed by a PR team within 10 weeks of symptom onset [11]. However, even in high income countries, despite fairly uniform facilitated access to health care, children with JIA are referred to PR centers with significant delay [12]. Some reasons are related to the disease itself: frequent insidious onset, and a long referral pathway including multiple specialists and unneeded procedures [13] contribute to the delay. A long median diagnostic delay has also been described for other pediatric chronic rare disease such as Crohn’s disease (10.1 months) [14], ulcerative colitis (5.8 months) [14] and lupus (2.8 months) [15].

There is a lack of published data about access to PR care for JIA patients from low and middle income countries [12]. India is the most populous low and lower middle income country worldwide with a population of 1.3 billion and 28.6% < 15 years of age [16]. There are no available epidemiological studies but the estimated number of Indian children with JIA ranges between 350,000 [17] to 1.3 million [18]. Management of these patients in low and middle resource countries is a global concern: recent recommendations about JIA management in less resourced countries (JAMLess) advocate that new patients with suspected JIA should be seen by a PRst within 4 weeks from the time of referral [19]. In India the delay to PR centers is likely to be more than in high income countries [20] because the shortage of pediatric rheumatologists (PRst) is more significant [21] and health-care costs are paid out of pocket by patients [22]. These parameters are likely to impact the referral pathways to PR centers but have not been systematically studied to date.

This is the first study which aims to describe the time from onset of symptoms to first PR visit for children with JIA to a tertiary center in India and to analyze factors that impact this.

## Patients and methods

This center systematically collects data on PR patients that covers the broad spectrum of JIA, connective tissue diseases and vasculitides. This retrospective cohort study is based on the data collected on all JIA patients seen at least twice over a five year period from 1st October 2013 to 30th September 2018 in the PR division, Institute of Child health, Sir Ganga Ram Hospital (SGRH) in New Delhi, a tertiary post graduate teaching hospital where both paid (private) and free (poor) patients are seen. Patients who were previously assessed by a PRst or for whom data were incomplete were excluded.

The Ethics Committee of Sir Ganga Ram Hospital, New Delhi approved both the study and the informed consent forms (ref EC/07/11/267).

### Data collection

At enrolment, the following data were captured on the JIA form by the PRst:

Demographic and general data including date of birth, sex, dwelling place, family history of inflammatory disease (at first or second degree), schooling, public or private consultation.

History of the disease including date of symptom onset, date of first visit to the PRst, specialty of the referral practitioner, date of diagnosis if previously done, specialty of the doctor who managed the child, previous anti-rheumatic drugs.

Clinical history captured joint pain, joint swelling, morning stiffness, heel pain, inflammatory back pain, fever, rash, macrophage activation syndrome (MAS) and uveitis. This clinical history was analyzed only for patients diagnosed in SGR hospital because of a possible bias of memorization (poor or incorrect recall) for patients previously diagnosed and treated.

Clinical examination findings including active joint count (AJC) (number of joints with swelling, tenderness or limitation of range of motion) presence of enthesitis, psoriasis, uveitis, fever, systemic rash or MAS.

Laboratory features including for each child erythrocyte sedimentation rate (ESR) at one hour and according to the clinical relevance anti-nuclear antibody (ANA), positivity of rheumatoid factor (RF) or HLAB27.

### Definitions

Time to first PR visit was defined as the time from the onset of symptoms to the first visit to a PR center. Time to diagnosis was defined as the time from the onset of symptoms to the diagnosis. If parents had forgotten the exact date of symptoms or diagnosis, the 15th of the month was taken.

The public consultation was defined as a free assessment by the PRst, the private one required fees.

Health care practitioner (HCP) specialty was classified as pediatrician (ped), general practitioner (GP), adult rheumatologist (adult rheum), orthopedic surgeon (ortho) and other. If patient self-referred he was classified as “self”.

Patients who were not previously diagnosed as JIA at first PR visit were classified as group A. Patients who were previously diagnosed and treated by a non PRst before the first PR assessment were classified as group B.

Each JIA patient was classified according to the International League of Associations for Rheumatology (ILAR) criteria second revision, Edmonton, 2001 based on number of joints involved, associated symptoms and laboratory features [23].

AJC was defined using the JADAS 71 score [24].

ESR was considered positive when its value exceeded 20 mm at the first hour [24], and ANA when titers exceeded 1:160.

Clinical remission was defined as the absence of signs and symptoms of inflammatory disease activity, including extra- articular manifestations [25].

Previous treatments were classified as nonsteroidal anti-inflammatory drugs (NSAIDs), steroids (oral, intra-muscular (IM), intra-venous (IV) or intra-articular (IA)), DMARDS, BRMs and alternative medicine.

The distance from patients dwelling place to the pediatric rheumatology center was calculated using an Internet-based route calculator, URL: https://www.google.com/maps/

### Statistical analysis

According to the objective of study no sample size estimation was performed. Moreover, the multivariable analyses were carried out according to rules-of-thumb reported in the literature concerning the minimum number of subjects required to conduct multiple regression analyses [26].

Patients from group B were already diagnosed appropriately, managed and treated prior to their first assessment in PR at SGRH and therefore they were excluded from the analysis of predictive factors that impacted the referral pathway. Indeed, longer duration of symptoms reduces the recall of history of the disease. Moreover, ongoing treatments would impact both the clinical examination and ESR.

Statistical analysis was done using SPSS Statistics for Windows, Version 17.0. Chicago: SPSS Inc. The median symptom duration prior to PRst visit in group A was 3.3 months. Thus, the cut off for early and late referral was chosen to be 3 months. Characteristics associated with early and late referral were examined. Pearson’s Chi square and univariate logistic regression was used to compare categorical variables with time to referral. Shapiro-Wilk test was used to assess normality of quantitative variables, and Mann-Whitney U-test applied for comparison in various groups. ROC curve was used to define the cut-off value of distance from home to hospital associated with longer time to PR referral. The multivariate logistic regression model included all the variables with a *p* value < 0.05.

## Results

### Characteristics at first PR visit

**Fig. 1:**
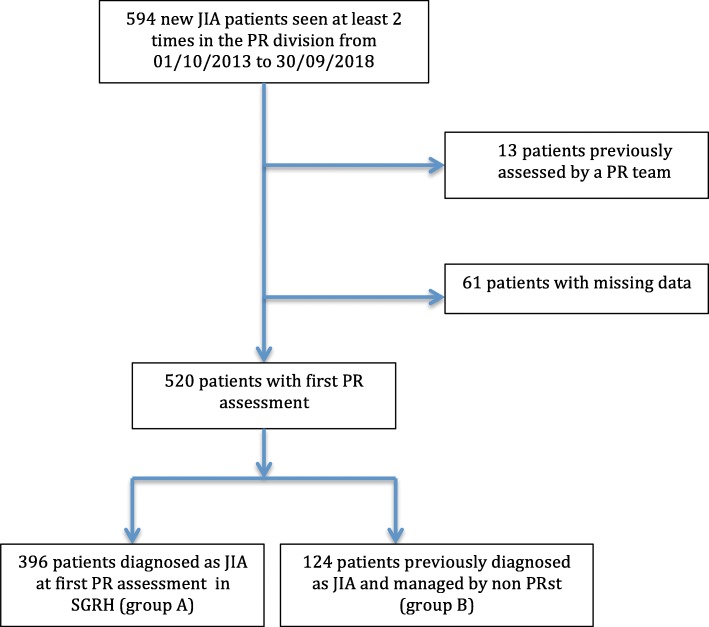
Flow chart for patients inclusion. PR: pediatric rheumatology, JIA: juvenile idiopathic arthritis, SGRH: Sri Ganga Ram Hospital, PRst: pediatric rheumatologist

A total of 520 out of 594 eligible new JIA patients were included: 396 (76.2%) in the group A, 124 (23.8%) in the group B (Fig. 1).

Median age at first PR visit was 10.0 years, 45.2% were female (Table 1). The most frequent JIA subtype was enthesis related arthritis (ERA) (47.5%), of which 86.1% were HLA B27 positive. Among ERA patients from group A, 39.7% had an history of fever preceding or associated with joint pains. Systemic JIA (sJIA) was the second most common subtype (23.8%). Half of the oligoarticular JIA patients (oJIA) had positive ANA. RF were positive in 42.6% of the polyarticular JIA patients (pJIA). Only 45 patients (8.7%) were assessed in the free system.

**Table 1:** Patient’s characteristics at first presentation to PR center

	All JIA ***n*** = 520 (100)	ERA ***n*** = 247 (47.5)	sJIA ***n*** = 124 (23.8)	pJIA ***n*** = 69 (13.3)	oJIA ***n*** = 68 (13.1)	undJIA ***n*** = 10 (1.9)	psoJIA ***n*** = 2 (0.4)
Age at first PR visit	10.0 [5.5, 13.2]	12.4 [10.1, 14.7]	6.2 [3.9, 9.9]	8.3 [4.2, 11.8]	5.1 [2.6, 8.6]	7.2 [5.2, 16.0]	6.5 [1.3, 11.6]
Female sex (%)	235 (45.2)	62 (25.1)	59 (47.6)	52 (75.4)	53 (77.9)	7 (70)	2 (100)
**Median time from symptoms onset to 1st assessment in PR (months)**							
Whole cohort	4.1 [1.8, 15.6]	4.1 [1.5, 17.5]	3.8 [1.5, 11.0]	6.2 [2.8, 18.4]	4.6 [2.5, 14.9]	8.3 [2.2, 21.4]	1.2 [0.6, 1.7]
Group A	3.3 [1.4, 10.2]	3.1 [1.2, 12.4]	2.4 [1.3, 6.4]	3.5 [1.9, 10.4]	3.9 [2.1, 9.5]	8.3 [2.2, 21.4]	1.2 [0.6, 1.7]
Group B	13.8 [3.8, 34.5]	11.5 [3.4, 30.8]	13.1 [3.7, 50.0]	18.4 [7.5, 44.8]	19.0 [4.2, 33.8]	0	0
**Median time from symptoms onset to diagnosis (months)**							
Whole cohort	3.3 [1.5, 11.2]	3.1 [1.3, 13.7]	2.4 [1.2, 6.5]	4.1 [2.3, 12.3]	3.9 [2.0, 10.7]	8.3 [2.2, 21.4]	1.2 [0.6, 1.7]
Group A	3.3 [1.4, 10.2 ]	3.1 [1.2, 12.5]	2.4 [1.3, 6.1]	3.5 [1.9, 10.4]	3.9 [2.1, 9.5]	8.3 [2.2, 21.4]	1.2 [0.6, 1.7]
Group B	4.3 [1.5, 15.8]	3.1 [1.4, 17.5]	2.7 [1.0, 9.5]	7.1 [3.8, 17.0]	4.6 [1.2, 15.2]	0	0
**Patients with time from symptoms onset to 1st PR visit <10 weeks (as per as BSPAR guidelines)**							
Whole cohort	160 (30.8)	78 (31.6)	47 (37.9)	14 (20.3)	16 (23.5)	3 (30)	2 (100)
Group A	145 (36.6)	71 (37.4)	41 (45.6)	13 (27.1)	15 (26.8)	3 (30)	2 (100)
Group B	15 (12.1)	7 (12.3)	6 (17.6)	1 (4.8)	1 (8.3)	0 (0)	0 (0)

Data are presented as frequencies (associated percentages) or as median [interquartile range]

*JIA* juvenile idiopathic arthritis, *oJIA* oligoarticular JIA, *pJIA* polyarticular JIA, *sJIA* systemic JIA, *ERA* enthesitis related arthritis, *psoJIA* psoriatic JIA, *UndJIA* undifferentiated JIA, *PR* pediatric rheumatology, *BSPAR* british society for paediatric and adolescent rheumatology standards of care

### Symptom duration

Median time to first PR visit was 4.1 months. Median time to diagnosis for the whole cohort was shorter than median time to first PR visit (3.3 versus 4.1 months) since group B patients had been previously diagnosed at the time of first assessment in PR. One hundred and sixty children (30.8%) were assessed by a PRst within 10 weeks after onset of symptoms. The shortest time to PR visit was for children presenting with psoriatic JIA (psoJIA) (median 1.2 months) however there were only 2 children in this subtype. The second shortest time was for sJIA patients (median 3.8 months). The longest time was in those presenting with undifferentiated JIA (undJIA) (median 8.3 months).

The time from onset of symptoms to diagnosis was also the shortest for children presenting with pso JIA (median 1.2 months), followed by sJIA (median 2.4 months). The longest time was in those presenting with undJIA (median 8.3 months).

### Differences between children from group A and group B

Patients from group B had a longer time to diagnosis than patients from group A (4.3 months versus 3.2 months) but it did not reach any statistical significance (Table 2). The median time from diagnosis to first PR visit was 3.6 months in group B and 0 in group A.

**Table 2:** Comparison between patient’s characteristics at first presentation to PR center

	Patients diagnosed as JIA at first assessment in PR center (group A, ***n*** = 396)	Patients diagnosed as JIA before first assessment in PR center (group B, ***n*** = 124)	***p*** value
Age at onset (years)	8.7 [4.2, 11.8]	9.3 [5.4, 12.1]	NS
Age at diagnosis (years)	9.7 [4.9, 12.8]	10.0 [6.1, 12.7]	NS
Median time from onset of symptoms to first assessment in PR center (months)	3.3 [1.4, 10.2]	13.8 [3.8, 34.5]	< 0.001
Median time from onset of symptoms to diagnosis (months)	3.2 [1.4, 10.2]	4.3 [1.5, 15.8]	NS
Median time from diagnosis to first assessment in PR center (months)	0	3.6 [1.2, 19.3]	< 0.001
Musculoskeletal features			
*AJC*	3 [1, 6]	4 [2, 8]	< 0.01
*Hip arthritis*	94 (23.7)	28 (22.6)	NS
*Cervical involvement*	21 (5.3)	13 (10.5)	< 0.05
ESR (mm/h)	51.0 [26.0, 82.0]	42.5 [21.3, 63.8]	< 0.05
Ongoing treatment			
*NSAIDS*	125 (31.6)	60 (48.4)	< 0.001
*Corticosteroids (oral, IV, IM)*	44 (11.1)	75 (60.5)	< 0.001
*Intra articular steroids*	1 (0.25)	15 (12.1)	< 0.001
*DMARDS*	0 (0.0)	102 (82.3)	< 0.001
*Biologics*	0 (0.0)	9 (7.3)	< 0.001
*Alternative medicine*	11 (2.8)	2 (1.6)	NS
Non attending to age appropriate school	36 (9.1)	20 (16.1)	< 0.05
Referral (group A) or followed (group B)			
*Ped*	198 (50)	24 (19.4)	< 0.001
*Adult rheum*	75 (18.9)	82 (66.1)	< 0.001
*GP*	8 (2)	0 (0)	NS
*Ortho*	75 (18.9)	18 (14.5)	NS
*Self*	32 (8.1)	0 (0)	< 0,001
*Other*	8 (2)	0 (0)	NS
Median distance from the PR center (km)	79.6 [19.8, 422.3]	205.0 [39.0, 688.5]	< 0.001

Data are presented as frequencies (associated percentages) or as median [interquartile range

PR: pediatric rheumatology, JIA: juvenile idiopathic arthritis, AJC: active joint count, ESR:erythrocyte sedimentation rate, NSAIDS: nonsteroidal anti-inflammatory drugs, IV:intravenous, IM: intramuscular, DMARDS: disease-modifying anti-rheumatic drugs

At first assessment in PR patients from group B had higher AJC (4 versus 3, *p* < 0.01) and more cervical spine involvement (10.5% versus 5.3%, *p* < 0.05). They had received more medications (*p* < 0.001 for each kind of medication) but despite this only 4.8% were in remission. Sixteen percent did not attend age appropriate school versus 9.1% in group A (p < 0.05).

Patients from group B were mainly managed by adult rheum (66.1% versus 18.9% in group A, p < 0.001), less frequently by peds (19.4% versus 50%, p < 0.001) and they resided further away (205.0 km versus 79.6 km p < 0.001).

There was no difference regarding JIA for any subcategories between the two groups (*p* = 0.168).

### Referral pathway

For the entire cohort patients were mainly referred by ped (42.7%), adult rheum (30.2%) and ortho 17.9%. Few children were referred by GP (1.5%) or other adult specialists (1.5%). Six percent were not referred by any doctor and self-referred.

In each group the shortest time to first PR visit was for children referred by peds but it did not reach any statistical significance (Tables 3 and 4).

**Table 3:** Time from onset of symptoms to first assessment in PR (months)

	Group A	Group B
Ped	2.9 [1.3, 8.4]	6,3 [3.3, 27.3]
Adult Rheum	3.5 [1.8, 17.0]	15,6 [3.8, 34.1]
Ortho	4.4 [1.4, 11.7]	14,9 [4.4, 62.9]
GP	3,4 [1.7, 5.8]	0.0
Other	8.7 [1.7, 28.0]	0.0
Self	2.8 [1.2, 14.7]	0.0

### Distance

**Fig. 2:**
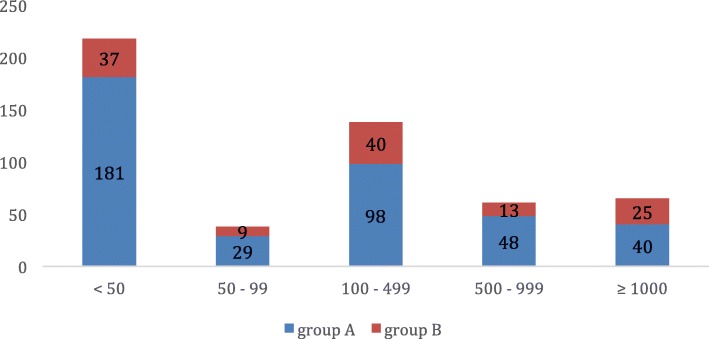
Travelling distance for each group. In abscissa number of patients, in ordinate distance in km

The median journey distance to the PR center was 119.5 km and IQR was very broad: [22.4, 465.0]. In group A, 40 patients (10.1%) travelled more than 1000 km, 25 (20.1%) in group B. The longer travelling distance, the higher proportion of patients from group B (Fig. 2).

### Predictive factors for the time to first PR visit prior to 3 months

Assessment in the private system, a history of any rheumatological disease in the family, fever or acute uveitis with red eye were significantly associated with a shorter time before the first PR visit. A longer distance or a lower ESR were significantly associated with a longer time before the first PR visit. There was a trend towards those children with a longer symptoms duration and a higher AJC (*p* = 0.06) or a history of rash (*p* = 0.09), although this did not reach statistical significance (Table 4). There was no statistical difference regarding JIA subtypes, the referral source or site of inflammatory joint disease (upper limb, lower limb, hip, cervical or temporomandibular joint involvement).

**Table 4:** Association between patients characteristics and symptoms duration at first PR assessment

	Symptoms < 3 months	Symptoms >or = 3 months	*p* value
Sex female	89 (47.3)	88 (42.3)	NS
Age at first PR visit	9.4 [4.4, 12.4]	10 [5.2, 13.1]	NS
Private OPD	177 (94.1)	183 (88.0)	< 0.05
Clinical examination			
*AJC*	2.5 [1, 5]	3 [1, 7]	NS
*LROM*	73 (38.8)	97 (46.6)	NS
History of:			
*Familial history of inflammatory disease*	38 (20.2)	22 (10.6)	< 0.05
*Joint pain*	174 (93.5)	202 (96.2)	NS
*Swelling*	153 (82.3)	187 (89)	NS
*Fever*	95 (50.5)	81 (38.9)	< 0.05
*Rash*	34 (18.1)	25 (12)	NS
*MAS*	5 (2.7)	4 (1.9)	NS
*Heel or tibial tuberosity pain*	13 (6.9)	16 (7.7)	NS
*Inflammatory Back pain*	47 (25.3)	53 (25.2)	NS
*Morning stiffness*	62 (33)	74 (35.7)	NS
*Acute uveitis (red eye)*	14 (7.4)	6 (2.9)	< 0.05
*Chronic uveitis (white eye)*	25 (13.3)	38 (18.3)	NS
Median distance with the PR center	36.6 [17.6, 263.3]	167.5 [25.6, 529.5]	< 0.001
Median ESR (mm/h)	64 [34, 90]	40 [22.5, 71]	< 0.001

Data are presented as frequencies (associated percentages) or as median [interquartile range

PR: pediatric rheumatology, OPD: out patient department, AJC: active joint count, LROM: limitation of range of motion, MAS: macrophage activation syndrome, ESR: erythrocyte sedimentation rate

**Fig. 3:**
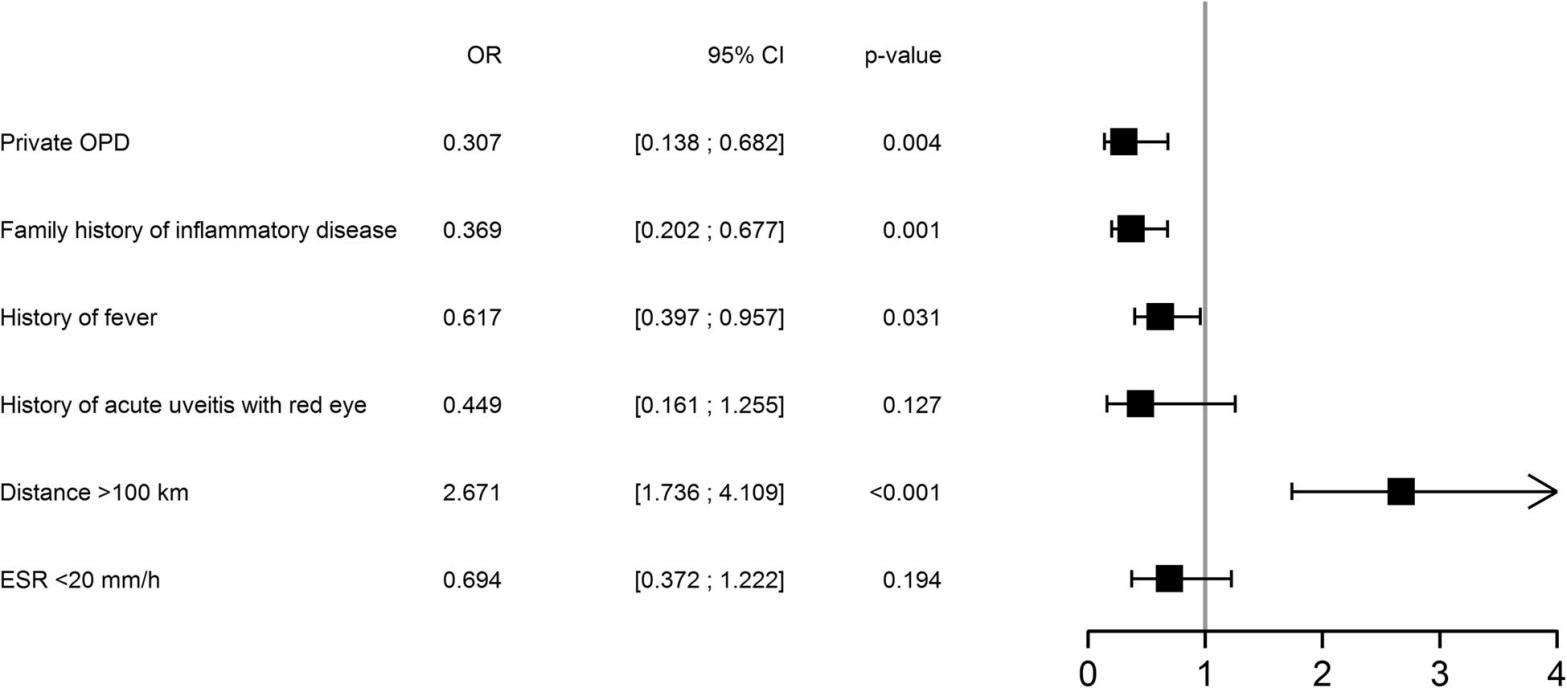
Multivariate analysis of predictive factors of time to first PR visit. OR:odds ratio, CI confidence interval, OPD: out-patient department, ESR: erythrocyte sedimentation rate

Using a multivariate logistic regression model the presence of a history of inflammatory disease in the family, history of fever, travelling a distance less than 100 km or consulting in the private system remained independent factors associated with being assessed within 3 months from symptoms onset (Fig. 3).

## Discussion

The median time to first PR visit at this Indian center was 4.1 months for the whole cohort and 3.3 months for group A. This is fairly comparable to the time described in high income countries: 3 [27] and 3.3 [28] months in France, 3 months in Germany [29], 3.8 months in Canada [30], 5.5 months in the UK [31] and 10 months in the United Arab Emirates [32]. In the EPOCA study the interval from onset to referral was 4.8 months in Africa and Middle East and 7.2 months in Southeast Asia [10]. This is similar to adult inflammatory rheumatic disease referral patterns where, a Danish registry, reported a median diagnostic delay in patients with rheumatoid arthritis, psoriatic arthritis and ankylosing spondylitis of 5.7 months [33]. In this Indian cohort 30.8% of the children met the British guidelines of being assessed in a PR center within10 weeks from symptom onset [11]. Here again, this is in line with what was previously described in a study from UK [34] and France where 26 and 45% [28] of the patients were compliant with the guidelines.

These results are surprising because previous studies such as EPOCA study showed that patients living in countries with lower gross domestic product (GDP) including India, had greater disease activity and damage than those living in wealthier countries [10]. Damage was associated with referral delay. Moreover Indian global health indicators are poorer than in high income countries: in India the probability of dying under 5 years of age (per 1000 live births) is 39, versus 4 in France or 7 in the USA [35]. The life expectancy at birth (in years for male/female) is 67/70 versus 80/86 in France or 76/81 in USA. The total expenditure on health per capita is 16 dollars in India [36], 9403 in USA and 4508 in France [35]. Health expenditure counts for only 1.02% of the Indian GDP [36]. There is no universal free access to health care. The health care is primarily delivered by the private medical sector which represents 80% of total expenditure on health. The majority (80%) of doctors work in cities where only 30% of the population resides [35]. This results in deep social health inequalities as corroborated by our study where being assessed in the private system was a univariate and independent factor of a shorter time to first PR visit. As the majority of patients were seen in the private sector we can assume that our delay was rather short and that our results are not applicable to the whole country. Data comparing private and public sector in India are scarce however a study found that private patients were seen for longer durations, were more likely to have a physical exam and their diagnosis explained than in public sector [37]. Analysis in several low and middle income countries suggested that private sector offers shorter waiting periods, more flexible opening hours and better availability of doctors [38]. In this PR center, all patients with a referral letter are seen within two-three days following the request for an appointment. In comparison, the European Union project Single Hub and Access point for PR in Europe (SHARE) survey which aimed to describe the current organization and delivery of specialist PR care across 29 European and allied countries estimated that nearly 70% of new patients were seen within 8 weeks of referral [39]. In a UK cohort, the median time between referral and assessment was 4 weeks [IQR 1.3, 8] and only 52.9% of the patients followed the BSPAR guideline and the JAMLess recommendations of being assessed within 4 weeks of the referral letter [31] versus 100% in our center. This reduces significantly the total delay to the first PR visit.

Furthermore, this center has both a local and a national presence in the area of PR and organises several updates in this specialty in New-Delhi and in distant cities as well. Thematic days such as « World arthritis day », « Children’s day », « World lupus day» are organized for the patients and their families on an annual basis. This helps to exchange thoughts and experiences and draw succour from each other regarding the disease and its consequences in daily life. The department is well publicised on internet by a proper website explaining the broad spectrum of rheumatological disorders and the activities of the medical team [40]. All these factors increase the visibility of the department and awareness about pediatric rheumatic diseases for both the medical community and patients which likely contributes to facilitate referrals and reduce delay to reach this PR center. Moreover, patients who can afford the private fees belong most frequently to the growing middle class in which increasing education and awareness is inducing a greater demand for better health care [18].

In comparison to high income countries, the high prevalence of adult rheum in the care pathway has to be pointed out. Indeed, adult rheum referred 30.2% of the cohort versus less than 6% in a UK study [41] or 7% in a French one [28]. In the European SHARE survey which included both Western and Eastern European countries, less than 16% of children were managed by adult rheum [39]. In the United Arab Emirate the adult rheum was implied in 70% of patients pathway [32]. In this country, similar to India, the number of PRs is limited. As there are only a handful of trained PRst in India (less than 15 for a population of 1.3 billion versus 1.1 PR per 1 million general population in Europe [39]), the current health care delivery has to be a joint journey with the adult rheum for the majority of patients [22]. However the number of adult rheum is also low with 0.02 Indian adult rheum per 100,000 versus 3.80 per 100,000 in France [21]. Thus, as described in Group B, Indian JIA patients are frequently diagnosed and treated by doctors without specific training in rheumatology such as peds or orthos. In this group despite ongoing treatments at first PR assessment, patients had a more aggressive disease with higher AJC, more cervical involvement, high ESR and more inappropriate schooling. Only 4.8% were in remission whereas the median disease duration was 13.8 months (IQR [3.8, 34.5]) which is not in line with international recommendations advocating clinical remission within 6 months following treatment onset [25]. Facing this common problem of shortage of PRst in low and middle countries, JAMLess recommendations advocate that in the absence of PRsts, patients should be assessed by clinicians knowledgeable and skilled in caring for children with rheumatic disorders who, ideally, are affiliated with an established rheumatology clinical network [19]. However Spencer in a commentary has suggested that in countries where PRsts are not available, the help of other specialists is essential and appreciated but this is a stop-gap solution and development of PR in every country is the only acceptable long term solution [42].

As the number of PRst is very small, and the land mass of India large, many patients travel a long distance for medical care: the median distance was 119.5 km which is more than previously described from France (26 km) [28], Canada (38.2 km) [30] and in Germany 38.8 km [29]. In the SHARE study, only 20% of patients travelled more than 150 km to obtain access to specialist PR care [39]. Group B patients resided further away than group A patients and distance was both a univariate and independent factor for a longer time to first PR visit. Training programs in pediatric rheumatology which are now available at a few centers in India including this center are essential to provide good care countrywide and reduce patients’s journey [22]. Clinicians caring for patients with JIA should be encouraged to organize and participate in relevant educational activities [19]. As advocated in the SHARE survey, educating primary health care providers settled in areas far away from tertiary centers is definitively crucial for improving early referral and also building clinical networks and shared care to facilitate delivery of care closer to home [39]. Additionally, the recent JAMless publication [19] has suggested that a mandatory module on PR during the training of all medical doctors who could be involved in the care pathway of JIA patients should be introduced to improve awareness about rheumatological diseases and has pointed out the importance of referring the child early to a PRst [19]. Indeed, studies have shown that pediatricians are not confident about their skills to examine the musculoskeletal system and often do not assess joints as part of routine clinical assessment of their patients [43]. Thus, easy screening tools to detect musculoskeletal disease early, such as the British pediatric Gait, Arms, Legs, Spine screen (pGALS) [44] or other previously published tools [45] should be used on a regular basis to screen children with musculoskeletelal complaints and therefore appropriately refer them. International collaborations with established PR centers in Europe, Canada and the USA are needed to develop world class centers of excellence in management and research in India where the large patients pool available offers a prospect for excellent research projects and studies [18]. The collaboration with the Paediatric Rheumatology International Trials Organisation (PRINTO), Paediatric Rheumatology European Society (PReS) and Childhood Arthritis and Rheumatology Research Alliance (CARRA) also facilitate and coordinate clinical trials and research in PR in India. The Indian Academy of Pediatrics has recognized PR as a specialty in 2001 [22]. This has promoted the development and the recognition of the specialty, however efforts have to be sustained to provide quality PR care India wide.

In comparison to other cohorts, there are significant differences regarding JIA subtypes: after exclusion of the 2 psoJIA patients, sJIA patients had the shortest time to PR referral. This has already been described earlier [27–29, 41]. Indeed the presence of fever which is [23] and will remain [46] a specific mandatory criteria for the diagnosis of this JIA subtype leads to a prompt consultation with a health care practitioner [13]. In this study fever was both a univariate and independent factor of a shorter time to first PR visit. However in other studies the time to PR visit of sJIA patients was shorter: 0.5 month in France [28], 1.1 month in Germany [29] and 1.3 month in UK [34]. Another Indian study confirms this rather long delay with a median diagnostic delay of 6 months [20]. This can be explained by the high incidence of infectious diseases in India that can present with prolonged pyrexia sometimes associated with articular symptoms or rash. Facing a child with fever and chronic arthritis practitioners consider tuberculosis [47], reactive arthritis (especially post streptococcal or Poncet’s disease), HIV or other viral infections prior to sJIA in the differentials [19, 48]. A clinical trial with antibiotic therapy against TB and/or streptococcus is frequently started and the child is reassessed after several weeks to months at the end of the antibiotics course which tends to increase the time to referral of sJIA patients to the PRst.

On the contrary, time to first PR visit of ERA patients was shorter than previously described (3.1 months in group A versus 11.4 months in a French study [28]). It has already been observed that in Asia, at disease onset ERA patients frequently have fever, swollen and painful joints in addition to high ESR and CRP value [49]. This acuity of symptoms can explain the short delay to first PR visit [12]. Acute uveitis and family history of inflammatory disease are also more frequently described in this JIA subtype and this study is the first to prove their association with a shorter delay to PR visit as univariate factors. Family history of inflammatory disease remained an independent factor as well: a family with a previous history of rheumatological disorders will be more aware about JIA symptoms. Acute uveitis causes red and painful eye that leads to a quick assessment by health care provider. As described in EPOCA study there is wide variability in the prevalence of JIA subtypes across geographical areas with a greater prevalence of sJIA and ERA and less ocular involvement in southeast Asia which might be related to different genetic determinants and perhaps environmental triggers [10]. In India, the most common JIA subtype is ERA [50, 51]. Thus, Indian practitioners have possibly a better knowledge of this JIA subtype which tends to reduce the time spent in the care pathway.

Important additional information from this study is that females were not referred later than males. Indeed, India is a country with documented culturally ingrained parental preference for sons inducing less investment in girls’ health and education [16, 52]. This preference is less important in urban and educated population [16] which may explain the gender equality regarding the delay to first PR visit in this cohort.

As a continuous variable a low ESR was associated with a longer delay as it has been previously described [28, 41] suggesting that a diagnosis of inflammatory arthritis may not be considered in the setting of normal inflammatory markers. However, this did not remain in the multivariate analysis in which we chose the cut off of 20 mm/h to be in line with the international classification [24]. Of note however in a developing country, with a high prevalence of iron deficiency [53] and infectious diseases ESR is frequently higher than 20 mm/h without any inflammation so this cut off is possibly not entirely valid for the subcontinent.

Previous studies from India have described a poor outcome of children with JIA [54, 55] and suggested that a long delay to diagnosis is part of the problem [20]. However we have demonstrated a rather short referral time: an important limitation of our study is that our population is not a representative sample of all Indian children with JIA for several reasons: First, data were collected in a private tertiary center in New Delhi, the capital city of India where most patients are from middle or high socio professional category with a better awareness and financial resources. It would have been very informative to capture parents’ socio professional category which influences time to referral with a probable prompt response in families with a higher level of education [30, 41, 56]. Secondly, this data set studied only JIA patients seen at least twice by PRst: proper epidemiological studies including JIA patients from rural and non rural areas whatever the subspecialty of the doctor would be more representative. Finally there is a likely referral bias as it can be presumed that complex patients (difficult to treat) are more frequently referred for a second advice to the PRst.

There are additional limitations of this data set: the date of symptom onset was defined by the parents, so a memory bias cannot be excluded. The entire patient’s pathway of care (number of HCP, dates of appointments, non-appropriate investigations or treatments such as antibiotics) and the presence of an ophthalmologic screening before first PR visit were not available.

## Conclusions

This is the first study about access to PR care in India. Time to first PR assessment at this center is comparable to high income countries. Cost of care and long distance to travel delayed consultation whereas acuity of complaints and a family member with rheumatologic condition hastened referral.

Possible factors to improve referral to PR centers would be to increase the number of PRst, to improve the training of health care practitioners regarding rheumatologic conditions especially for those who practice in remote locations far away from tertiary centers and to improve medical insurance coverage. Indeed many families can ill afford the travel and cost of care for a child with JIA.

The power of low-priced easy access for both digital technologies and bio similars is waiting to be harnessed to improve the lives of many Indian children with JIA. It is the need of the hour to have trained personnel on the ground and have referral pathways defined for core symptoms of JIA that enables fast tracking of children with inflammatory joint disease for urgent care.

## Data Availability

The datasets used and/or analysed during the current study are available from the corresponding author on reasonable request.

## Abbreviations

adult rheum: adult rheumatologist
AJC: active joint count
ANA: anti-nuclear antibody
BRMs: biologic response modifiers
BSPAR: British Society for Pediatric and Adolescent Rheumatology Standards of Care
DMARDS: disease-modifying anti-rheumatic drugs
ERA: enthesis related arthritis
ESR: erythrocyte sedimentation rate
GDP: gross domestic product
GP: general practitioner
HCP: health care practitioner
IA: intra-articular
IM: intra-muscular
IQR: interquartile range
IV: intra-venous
JIA: juvenile idiopathic arthritis
MAS: macrophage activation syndrome
NSAIDs: nonsteroidal anti-inflammatory drugs
oJIA: oligoarticular JIA patients
ortho: orthopedic surgeon
ped: pediatrician
polyJIA: polyarticular JIA
PR: pediatric rheumatology
PRst: pediatric rheumatologist
psoJIA: psoriatic JIA
RF: rheumatoid factor
SGRH: Sri Ganga Ram Hospital
sJIA: systemic JIA
SRI: self- relative- internet
undJIA: undifferentiated JIA
